# Functional additives in fishmeal-replaced aquafeeds: precision strategies for sustainable aquaculture

**DOI:** 10.1186/s40104-026-01463-2

**Published:** 2026-07-13

**Authors:** Weidan Jiang, Pei Wu, Hongyun Zhang, Yaobin Ma, Yang Liu, Xiaoqiu Zhou, Lin Feng

**Affiliations:** 1 Fisheries College, Sichuan Agricultural University, Chengdu, Sichuan 611130 China; 2 Key Laboratory for Animal Disease-Resistance Nutrition of China Ministry of Education, Sichuan Agricultural University, Chengdu, Sichuan 611130 China; 3 Aquatic Health and Intelligent Aquaculture Key Laboratory of Sichuan Province, Chengdu, 610093 China

**Keywords:** Amino acid, Aquaculture, Enzyme preparation, fishmeal replacement, Functional additive, Probiotic, Protein resource

## Abstract

Aquaculture serves as a critical pillar of global food security and human nutrition by providing high-quality protein, essential fatty acids, and efficient feed conversion while minimizing land use. However, its rapid expansion depends heavily on stable supplies of high-quality protein sources, particularly fishmeal (FM), which faces growing instability due to climate change and overfishing. While sustainable alternative protein sources like plant and insect proteins have been explored, high inclusion levels often impair growth and health in aquatic animals, partly due to poor palatability, amino acid (AA) imbalances, low digestibility, and antinutritional factors (ANFs). Precision strategies, especially functional additives, offer a promising solution by targeting these challenges: attractants enhance palatability, crystalline AAs correct AA imbalances, exogenous enzymes improve digestibility, and functional compounds mitigate health impairments. Although research on functional additives for FM replacement lags behind studies on alternative protein sources, theoretical and experimental evidence confirms their potential. Precisely formulated functional additives not only counteract the drawbacks of alternative protein sources or enable higher substitution ratios, underscoring their innovative role in sustainable aquafeed development. This review highlights the targeted use of functional additives to address appetite suppression, AA imbalances, digestive inefficiencies, and health risks, offering actionable insights for researchers, aquaculture nutritionists, and the feed industry to advance both scientific knowledge and practical FM replacement solutions.

## Introduction

Aquaculture is essential for global food security and human health [1]. It developed rapidly to meet growing demand after wild fisheries reached their maximum sustainable yields in the 1980s [2]. The fast development of the aquaculture is highly dependent on high-quality protein. This is mainly because aquatic animals not only use proteins to build tissues, but also as a primary energy source, due to a limited capacity to utilize carbohydrates. Therefore, the protein requirements for aquatic animals, especially juveniles, are notably high (typically ranging from 30% to 50% of their diet), which is two to three times of the requirements for livestock (e.g., 18%−22% for piglets and growing pigs) and poultry (16%−23%) [3].

For decades, fishmeal (FM) has been the gold-standard protein in aquafeeds. Recently, global FM production reached about 3 million tons, with aquafeeds consuming roughly 90% of the supply [4]. However, FM availability has become increasingly unstable due to El Niño events and overfishing, leading to an approximately 40% decline in production between 2013 and 2023 [4]. This has resulted in resource scarcity, volatile prices, and supply uncertainty, driving global efforts to develop alternative protein sources [5]. However, high levels of alternative protein sources often impair growth and health of aquatic animals, partly due to palatability issues, AA imbalances, indigestible components, and ANFs. These problems may be particularly prominent in carnivorous fish. Therefore, strategically addressing these limitations has emerged as an important pathway toward sustainable aquaculture.

Functional additives offer a targeted strategy to overcome these problems associated with the use of FM-replaced aquafeeds [6]. While most plant-based or novel proteins do not fully replicate the nutritional profile of FM, their judicious application can effectively compensate for nutritional shortcomings and often enable higher inclusion levels. These additives function through precise intervention at specific points of limitation. First, feed attractants by releasing chemical cues detected by olfactory and gustatory systems, they stimulate feeding behavior, increase intake, and reduce nutrient leaching and waste. This is particularly important when plant-based proteins are introduced [7]. Second, crystalline AA can correct imbalances in the AA profiles of alterative proteins compared to FM. Supplementing limiting essential AA (EAA) ensures an ideal AA pattern is delivered, thereby supporting optimal growth, protein synthesis, and metabolic efficiency. Third, exogenous enzymes such as phytases, proteases, and carbohydrases degrade antinutritional factors like phytate and non-starch polysaccharides (NSP) present in plant protein sources. This action improves the digestibility of proteins, phosphorus, and carbohydrates while reducing nutrient excretion and water pollution. Fourth, functional compounds including nucleotides, organic acids, and prebiotics or probiotics support physiology beyond basic nutrition. They enhance gut health and microbiota balance, improve immune responses, mitigate inflammation induced by dietary stressors, and promote nutrient absorption. Those approaches transform the challenge of the use of alternative protein sources into an opportunity to develop more sustainable, cost-effective, and high-performing feed formulations through precision supplementation strategies.

This review provides a comprehensive examination and discussion on the strategic use of functional additives in FM-replaced aquafeeds. It evaluates the role of these additives in addressing key challenges associated with FM replacement. The analysis offers guidance for developing cost-effective, high-performance feeds and suggests partly directions for future research.

## Research methodology

This review was conducted through a systematic literature search across several academic databases and platforms, including Elsevier-ScienceDirect, Google Scholar, PubMed, Web of Science, and ResearchGate. The objective was to comprehensively identify relevant scientific articles published in English, primarily from 2010 to 2026. Approximately 70% of the retrieved literature was published from 2020 onward, though seminal earlier studies were also included when necessary. The search strategy utilized keywords encompassing the term ‘fishmeal replacement’, along with additional specific terms such as ‘feed attractant’, ‘crystalline amino acid’, ‘exogenous enzyme’, ‘pre/probiotics’, ‘taurine’, ‘betaine’, ‘(2-Carboxyethyl) dimethylsulfonium Bromide (Br-DMPT)’, ‘nucleotide’, ‘amino acid’, ‘lysine’, ‘methionine’, ‘tryptophan’, ‘threonine’, ‘xylanase’, ‘cellulase’, ‘phytase’, and ‘bile acid’. Studies were screened based on title, abstract and materials and methods for relevance to two core criteria: (1) the inclusion of a FM-based positive control, and (2) the efficacy of functional additives in addressing challenges associated with FM-replaced aquafeeds. For each section of the review, studies with FM positive controls are prioritized, and then studies without FM positive controls were excluded. However, it should be noted that where such studies are unavailable, a limited number of key representative studies were selected instead. Eligible full-text articles were thoroughly analyzed to synthesize evidence and evaluate the role of functional additives in supporting FM replacement strategies. From an initial pool of approximately 500 records, about 120 papers met the inclusion criteria and were selected for in-depth review.

## Innovative applications of functional additives in FM-replaced aquafeeds

Current FM alternative protein sources include animal by-products, plant proteins, insect meals (e.g., black soldier fly larvae, mealworms), single-cell proteins, and bioflocs [5, 8–10]. Functional additives are strategically incorporated to mitigate their limitations, as reviewed below.

### The application of feed attractants in FM-replaced aquafeeds

Feeding is how animals get nutrients and energy for normal growth [11]. Yet, replacing FM with alternative protein sources beyond certain levels often cuts feed intake (FI) in fish species, as seen with poultry by-product meal in juvenile large yellow croaker (*Larimichthys crocea*) [12], fermented soybean meal (SBM) in spotted seabass (*Lateolabrax maculatus*) [13], and bioflocs from integrated multi-trophic aquaculture in advanced juvenile red drum (*Sciaenops ocellatus*, Actinopterygii) [9]. The observed reduction in appetite can be partially explained by the abundance of potent feeding stimulants present in FM, which strongly promote feeding behavior. In contrast, alternative protein sources, particularly plant-derived protein sources, are typically deficient in these key feed attractants. It is well known that while taurine content in these plant meals is negligible, levels of other appetite-stimulating AAs (like glycine and alanine) in SBM or cottonseed meal (CSM) are only half of those found in FM [14, 15]. Therefore, incorporating exogenous feed attractants into diets formulated with alternative protein sources represents an effective strategy to mitigate this palatability issue.

Feed attractants are commonly classified by source into two categories, synthetically produced compounds and natural substances or extracts thereof. Among natural attractants, animal by-products are particularly significant, especially for carnivorous species. Study demonstrates their effectiveness, for instance, supplementing FM-replaced aquafeeds with jack mackerel meal enhanced FI and growth, even surpassing growth performance on the FM control diet in olive flounder (*Paralichthys olivaceus*) [16]. However, classifying feed attractants based on their specific type (rather than source alone) may provide a more useful framework for summarizing their application in FM-replaced aquafeeds. For this, feed attractants are primarily categorized into: (1) AAs and small peptides, (2) active methyl compounds, (3) nucleotides and nucleic acids, (4) lipids, and (5) plant extracts and others [11, 14].

Table 1 lists the application of feed attractants in FM-replaced aquafeeds. It can be seen that current research primarily focused on the first two categories, AAs and derivatives, and active methyl-containing compounds. Within the first category, taurine, glutamic acid, hydroxyproline, and AA mixtures are the main subjects of investigation. Researches show that supplementing certain AAs (e.g., taurine, hydroxyproline) can improve FI and growth in some fish species such as grass carp (*Ctenopharyngodon idellus*) [17] and Chinese perch (*Siniperca chuatsi*) [18] when replacing 5% or 15% FM with plant protein sources. However, similar benefits are not consistently observed across all aquatic species, such as shrimp (*Litopenaeus vannamei*) [19] or yellow river carp (*Cyprinus carpio* var.) [20]. Taurine has no effect on shrimp might be partly because replacing 10% FM with *Clostridium autoethanogenum* protein did not reduce FI or growth [19]. In the yellow river carp, the uncommon AA mixture (0.4% alanine, 0.5% arginine and 0.7% glycine) used as feed attractants might be one of the reasons for its poor effect [20]. Overall, the application of AAs and derivatives as feed attractants in FM-replaced aquafeeds still requires further exploration on a larger variety of fish species, especially on carnivorous fish with higher proportion of alternative protein sources in the feed.

**Table 1:** The application of feed attractants in low FM or FM-free aquafeeds

Species	IBW, g	Period, d	**FM levels, %**		Alternative protein sources	NC Vs. PC	**NC + Feed attractants**				References
			PC	NC			Feed attractants	Optimal levels	Compared with the NC group	Compared with the PC group	
AAs and small peptides, %											
Grass carp (*Ctenopharyngodon idella*)	256	60	5	0	SPC	FI: ↓7% PWG: ↓13%	Taurine	0.1	FI: ↑18% PWG: ↑32%	FI: ↑10% PWG: ↑15%	[17]
Chinese perch (*Siniperca chuatsi*)	13.85	56	46	31	Fermented SBM	FI: ↓2% PWG: ↓41%	Hydroxyproline	1	FI: ↑15% PWG: ↑64%	FI: ↑13% PWG: NS	[18]
Shrimp (*Litopenaeus vannamei)*	0.32	51	25	15	*Clostridium autoethanogenum* protein	WG: NS	Taurine	0.4	WG: NS	WG: NS	[19]
Yellow river carp (*Cyprinus carpio* var.)	12.81	50	14	0	Plant protein diet	FI: ↓12% PWG: ↓17%	Amino acid mixtures	0.4% alanine, 0.5% arginine and 0.7% glycine	FI: NS PWG: ↑7%	FI: ↓8% PWG: ↓11% (Partial relief)	[20]
Active methyl compounds, mg/kg											
Grass carp (*Ctenopharyngodon idella*)	216	60	4	0	SPC	FI: ↓13% PWG: ↓19%	Br-DMPT	260	FI: ↑24% PWG: ↑43%	FI: ↑8% PWG: ↑16%	[7]
Nile tilapia (*Oreochromis niloticus*)	19.84	56	35.5	0	SBM	FI: ↓12% FBW: ↓14%	Betaine	2,000	FI: ↑15% FBW: ↑19%	FI: NS FBW: NS	[21]
					SBM and CGM	FI: ↓5% FBW: ↓8%			FI: ↑8% FBW: ↑15%	FI: NS FBW: ↑6%	
Rainbow trout (*Oncorhynchus mykiss*)	12.69	54	65	35	SBM	FI: NS PWG: NS	Betaine	20,000	FI: NS PWG: NS	FI: NS PWG: NS	[22]
Mixed attractants and others											
Yellow river carp (*Cyprinus carpio* var.)	12.97	50	14	0	Plant protein diet	PWG: ↓27%	Br-DMPT, tangerine peel and yeast powder	0.06% Br-DMPT, 0.22% tangerine peel powder, and 0.75% yeast powder	PWG: ↑24%	PWG: ↓9% (Partial relief)	[23]
Red hybrid tilapia (*Oreochromis* sp.)	7.31	56	46.98	11.74	Corn and/or SPC	FI: NS PWG: NS	Betaine and dried basil leaves	0.5% betaine and 2% dried basil leaves	FI: NS PWG: NS	FI: NS PWG: NS	[24]
Largemouth bass (*Micropterus salmoides*)	13.44	56	45	27	SPC	PWG: ↓4%	Functional palatability enhancer	0.15%	PWG: ↑5%	PWG: NS	[25]

*PC* Positive control (FM control), *NC* Negative control (low-FM or FM-free), *IBW* Initial body weight, *FI* Feed intake, *FBW* Final body weight, *WG* Weight gain, *PWG* Percent weight gain, *SGR* Specific growth rate, *NS* No significant difference

The second category encompasses active methyl-containing substances, characterized by molecules containing methyl groups, such as Br-DMPT and betaine. These compounds function not only as active methyl donors but also as feeding stimulants. Research demonstrated that incorporating 260 mg/kg Br-DMPT into an all-plant protein diet for grass carp effectively counteracts the reduced FI, feed conversion efficiency, and growth caused by FM replacement [7, 26–30]. This effect, superior to that achieved with an FM-based diet, is mediated through the regulation of brain neuropeptide Y expression and digestive enzyme activity [7, 27]. Interestingly, adding 2,000 mg/kg betaine to the diet fully restored the suppressed FI and growth in juvenile Nile tilapia (*Oreochromis niloticus*) resulting from the replacement of 35.5% FM with SBM or a blend of SBM and corn gluten meal (CGM) [21]. However, supplementing with 20,000 mg/kg of betaine did not further increase the FI and growth of rainbow trout (*Oncorhynchus mykiss*) when 30% of FM replaced by SBM [22]. This is likely attributable to the fact that SBM replacement of FM had no impact on rainbow trout FI or growth. Therefore, future studies should design feeds incorporating higher levels of alternative protein sources, supplemented with active methyl compounds as feed attractants, particularly for carnivorous fish.

Other categories have fewer studies. However, these experiments have only examined the effects of adding nucleotides or cholesterol to FM-free or low-FM diets on the FI and growth of Chinese mitten crabs (*Eriocheir sinensis*) [31] and turbot (*Scophthalmus maximus* L.) [14], indicating their potential application in FM-replaced aquafeeds. Nevertheless, these studies lacked FM-PC groups, making it impossible to determine their actual effectiveness in FM-replaced aquafeeds, thus necessitating further research. Interestingly, a growing number of studies are focusing on the combined use of feed attractants. For instance, Fang et al. [23] found that combined feed attractants consisting of 0.06% Br-DMPT, 0.22% tangerine peel powder, and 0.75% yeast powder were more suitable for the plant-based protein diet of yellow river carp (*Cyprinus carpio* var.). Additionally, the commercialized functional palatability enhancer provided by Lucta Co., Ltd. (Guangzhou, China) can also completely alleviate the negative impact of replacing 18% FM with SPC on the growth of largemouth bass (*Micropterus salmoides*) [25]. However, supplementing with 0.5% betaine and 2% dried basil leaves did not further increase the FI and growth of red hybrid tilapia (*Oreochromis* sp.), while corn and/or SPC replaced 34.25% of FM had no significant effect on those indexes [24].

Overall, the strategy of adding feed attractants allows for the complete substitution of FM with plant-based protein in herbivorous grass carp (4%–5% FM) and omnivorous tilapia (35.5% FM), achieving performance comparable to the FM positive control group. This approach also boosts the FM replacement rate in carnivorous fish (Chinese perch and largemouth bass) by about 15%–18%. Future research should investigate feed attractants in aquafeeds across diverse aquatic species, explore other methyl compounds within broader ranges of alternative protein sources and animals, and expand studies on combined feed attractants to improve the palatability and growth efficacy of FM-replaced aquafeeds.

### The application of crystalline AA and analogues in FM-replaced aquafeeds

Aquatic animals require approximately 10 EAAs, among which lysine (Lys), methionine (Met), tryptophan (Trp) and threonine (Thr) are the four primary ones requiring consideration. FM is valued as a high-quality protein in fish nutrition, because it offering high protein content, a balanced AA profile, and excellent digestibility [32]. However, alternative protein sources, particularly plant-based ones, often exhibit AA imbalances. For instance, soybean-derived products are deficient in Met, and similarly, as the proportion of CSM used to replace FM increases, the levels of Lys, Met, and Thr in the feed gradually decline below the required thresholds, indicating that replacing FM with CSM leads to deficiencies in these AAs [33]. Thus, supplementing with exogenous crystalline AAs may serve as one effective solution to address AA imbalance arising from FM-replaced aquafeeds. Currently, most studies on FM replacement, have supplemented EAA to meet AA requirements, especially Lys, Met, Trp and Thr. Additionally, some studies have examined the additional effects of incorporating crystalline AAs, including monomeric AAs and AA mixtures during FM-replaced, with relevant studies summarized in Table 2. These studies reveal that the most extensively investigated monomeric AAs in FM-replaced aquafeeds are Lys and Met, whereas Trp has been scarcely studied.

**Table 2:** The application of crystalline AAs in FM-replaced aquafeeds

Species	IBW, g	Period, d	FM levels		Alternative protein sources	**NC vs. PC**	**NC + Crystalline AAs**				References
			PC	NC			Crystalline AAs	Optimal levels, %	Compared with the NC group	Compared with the PC group	
Lys											
Rainbow trout* (Oncorhynchus mykiss,* Walbaum*)*	23.61	65	46.37	20	Wheat gluten meal	PWG: ↓23%	Lys	0.58	PWG: ↑55%	PWG: ↑19%	[34]
African catfish (*Clarias gariepinus*, Burchell 1822)	6.23	56	40	20	Slaughterhouse poultry by-product meal	PWG: ↓14%	Lys	1	FBW: ↑4%	FBW: NS	[35]
Chinese sucker (*Myxocyrinus asiaticus*)	3	56	60	42	CSM	SGR: ↓16%	Lys	0.65	SGR: ↑17%	SGR: NS	[36]
Meagre (*Argyrosomus regius*)	36	60	45	20	PP blends^1^	FBW: ↓19%	Lys	1.1	FBW: ↑15%	FBW: ↓18% (Partial)	[37]
Nile tilapia (*Oreochromis niloticus* L.)	1.93	70	20	0	SBM	/	Lys	0.5	/	PWG: ↑51%	[38]
Blunt snout bream (*Megalobrama amblycephala*)	107.9	56	5	0	RPC	PWG: ↓23%	Microcapsule Lys/Crystalline Lys	0.43/0.23	PWG: ↑9%	PWG: NS	[39]
Met											
Southern catfish (*Silurus meridionalis)*	19.73	56	57.0	27.3	SBM	SGR: ↓14%	Met	0.21	SGR: ↑21%	SGR: NS	[40]
Rainbow trout (*Oncorhynchus mykiss)*	106	90	50.63	0	SPC	FBW: ↓35%	Met	0.42	FBW: ↑16%	FBW:↓25% (Partial)	[41]
Chinese sucker (*Myxocyrinus asiaticus*)	13	56	40	16	SBM	SGR: ↓21%	Met	0.3	SGR: ↑10%	SGR: ↓13% (Partial)	[42]
Largemouth bass* (Micropterus salmoides)*	16.6	56	65.3	14.5	Mixture of feedstuffs^2^	PWG: ↓12%	Met	0.5	PWG: ↑36%	PWG: ↑20%	[43]
Gibel carp (*Carassius auratus gibelio)*	6.7	70	15	5	Plant proteins (SBM, RSM, CSM)	SGR: ↓9%	Met	0.1	SGR: ↑8%	SGR: NS	[44]
White shrimp* (Litopenaeus vannamei)*	3.08	56	26	10	Meat bone meal (4%), poultry by-product (4%) and SPC (11%)	PWG: NS	Met	0.3	PWG: ↑7%	PWG: NS	[45]
Trp											
Hybrid yellow catfish gut (*Pelteobagrus fulvidraco ♀* × *Pelteobagrus vachelli ♂*)	6.47	42	40	10	SBM	FBW: ↓14%	Trp	0.5/1.0	FBW: NS	FBW: NS Intestinal inflammation induced by SBM: ↓	[46]
AA Mixture											
*Argyrosomus regius*	32.4	84	38.5	11.0	CGM, Dehulled SBM	FBW: ↓46%	Taurine, Lys and Met	Taurine-0.5%, Lys-0.9% and Met-0.25%	FBW: ↑92%	FBW: ↑5%	[47]
							Taurine, Lys	Taurine-0.5%, Lys-0.9%	FBW: ↑75%	FBW: NS	
							Taurine and Met	Taurine-0.5%, Met-0.25%	FBW: ↑81%	FBW: NS	
							Lys and Met	Lys-0.9% and Met-0.25%	FBW: ↑57%	FBW: NS	
Red drum (*Sciaenops ocellatus*, Actinopterygii)	61.5	56	27.8	13.9	Biofloc meal	FBW: ↓65%	AA mixture	Leu0.6%, Lys0.8%, Thr0.3%, Met0.3% and Arg0.6%	FBW: ↑118%	FBW: NS	[48]

*PC* Positive control (FM control), *NC* Negative control (low-FM or FM-free), *IBW* Initial body weight, *FBW* Final body weight, *PWG* Percent weight gain, *SGR* Specific growth rate, *NS* No significant difference

^1^Plant protein (PP) blends: soybean, wheat, rapeseed, corn gluten and wheat gluten

^2^Mixture of feedstuffs: 45% poultry byproduct meal, 30% soybean meal, 15% blood meal, and 10% krill shrimp meal

Studies on Lys supplementation in FM-replaced aquafeeds have mainly examined alternative protein sources such as wheat gluten meal, CSM, SBM, rice protein concentrate (RPC), plant protein blends, and poultry by-product meal. Carnivorous fish, whose conventional diets often contain 40%–60% FM, show reduced growth when about 18%–26.37% of FM is replaced by plant or animal-based proteins. However, supplementing with 0.58%–1.0% Lys fully compensated for this decline, restoring growth to the level of FM-based diets in rainbow trout (*Oncorhynchus mykiss*, Walbaum) [34], African catfish (*Clarias gariepinus*, Burchell 1822) [35], and Chinese sucker (*Myxocyrinus asiaticus*) [36]. Interestingly, 1.1% Lys only partially alleviated the negative effects after replacing 25% FM with plant protein blends in Meagre (*Argyrosomus regius*) [37]. In addition, omnivorous and herbivorous fish depend less on FM, typically requiring only about 20% and 5% FM in their diets, respectively. For these species, such as juvenile Nile tilapia (*Oreochromis niloticus* L.) [38] and blunt snout bream (*Megalobrama amblycephala*) [39], adding around 0.5% Lys allowed complete FM replacement with plant proteins like SBM or RPC without compromising performance.

Met supplementation is primarily used when replacing FM with soybean-derived proteins such as SBM or SPC, or in mixed-protein diets containing soybean protein. Undoubtedly, replacing half or all FM with a single soybean protein source consistently reduced growth in carnivorous fish. While adding 0.21%–0.42% Met could fully counterbalance the negative effects of replacing about half of FM in species like southern catfish (*Silurus meridionalis*) [40]. However, with higher replacement levels, Met only partially alleviated the growth impairment in rainbow trout (*Oncorhynchus mykiss*) [41], and Chinese sucker (*Myxocyrinus asiaticus*) [42]. Notably, when Met was added to mixed protein sources (including but not limited to soybean-based ingredients) used to replace FM, even at replacement rates exceeding a half, it could completely reverse the adverse effects. In some cases, growth performance may even surpass that of the FM group, as observed in largemouth bass (*Micropterus salmoides*) [43], gibel carp (*Carassius auratus* *gibelio*) [44] and White shrimp (*Litopenaeus vannamei*) [45]. These results imply that the growth reduction linked to high dietary soybean protein may not be due solely to Met deficiency, but could also involve other factors such as reduced appetite or enteritis. In addition, as aquaculture strives for cost-effectiveness, adding crystalline AAs in FM-replaced aquafeeds raises expenses, driving interest in low-cost AA analogs. In grass carp, methionine hydroxy analog (MHA) outperformed DL-Met in enhancing growth, disease resistance, and muscle quality, with Met-Met and betaine showing similar benefits [49–53]. Whether these AA analogs work equally well in FM-replaced aquafeeds for other species requires further study.

Beyond Lys and Met, studies have also explored Trp and mixed AA supplementation. In hybrid yellow catfish (*Pelteobagrus fulvidraco* ♀ × *Pelteobagrus vachelli* ♂), adding 0.5%–1.0% Trp alleviated SBM-induced intestinal inflammation when most of FM was replaced, though it did not improve growth [46]. Additionally, supplementation with AA mixtures (e.g., taurine, Lys, Met) have fully offset the negative effects of replacing more than half of FM with plant protein in species such as *Argyrosomus regius* [47] and red drum (*Sciaenops ocellatus*, Actinopterygii) [48]. These results once again demonstrate that that SBM substitution involves more than just Met deficiency.

In summary, while single Lys supplementation allows full FM replacement in omnivorous/herbivorous fish (e.g., Nile tilapia, blunt snout bream), carnivorous species achieve only half of FM replacement with Lys or Met alone. Further substitution in carnivores requires addressing appetite, gut health, and AA balance, alongside the need to explore cost-effective AA analogs in FM-free feeds.

### Application of exogenous enzymes in FM-replaced aquafeeds

The primary objective of supplementing exogenous enzymes is to improve the digestibility and absorption of FM-replaced aquafeeds, with applications centered on two core scenarios. First, exogenous enzymes are required when aquatic animals cannot endogenously secrete digestive enzymes to degrade ANFs or toxic/harmful compounds in feed ingredients. Second, they compensate for insufficient endogenous enzyme secretion: larval/juvenile fish have underdeveloped digestive systems, enzyme production declines with senescence, and adult fish often experience impaired enzyme secretion due to stress or disease.

#### Non-starch polysaccharidases (NSPases)

Exogenous NSPases are predominantly used to enhance nutrient utilization in FM-replaced aquafeeds, especially those formulated with plant protein sources. Plant-based ingredients contain indigestible ANFs, such as NSP, phytic acid, and gossypol, with NSP being the most problematic. Aquatic animals exhibit negligible or no endogenous intestinal enzyme activity capable of breaking down NSP [54, 55]. NSP exerts three major anti-nutritional effects: (1) As the main component of plant cell walls, NSP resists endogenous enzymatic degradation, trapping intracellular nutrients, reducing feed digestibility, and slowing nutrient release by inhibiting solute diffusion; (2) Its high viscosity impairs mechanical mixing of intestinal digesta, creating uneven chyme, blocking the transport of sugars, AAs, and fatty acids to the intestinal mucosa, and disrupting fat emulsification; and (3) Viscous NSP binds to digestive enzymes, inhibiting enzyme–substrate interactions. NSP exists in both insoluble (e.g., cellulose) and soluble (e.g., arabinogalactan, β-glucan) forms, and is abundant in common plant feedstuffs such as SBM, cottonseed meal (CSM), rapeseed meal, and sunflower seed meal [56, 57]. Thus, exogenous enzyme supplementation is a promising strategy to alleviate these limitations in FM-replaced aquafeeds (especially plant protein-based).

The key NSPases include xylanase, β-glucanase, and cellulase [57]. Xylanase and β-glucanase mainly target soluble NSP to reduce intestinal viscosity, which strengthens the contact between feed particles and digestive enzymes, accelerates nutrient diffusion to the intestinal mucosa for enhanced absorption, and ultimately improves feed efficiency [58]. Cellulase (defined as β-1,4-glucan-4-glucan hydrolase) is a synergistic multi-enzyme complex (not a single enzyme), primarily consisting of exo-β-glucanase, endo-β-glucanase, β-glucosidase, and often highly active xylanase; it synergistically hydrolyzes insoluble cellulose into glucose [59]. Numerous studies confirm that single or combined supplementation of these enzymes in plant protein-rich diets effectively enhances feed digestibility, improves intestinal health, and promotes growth performance in fish. Examples include xylanase supplementation in juvenile Jian carp (*Cyprinus carpio *var*.* Jian) and on-growing grass carp [60, 61], and a xylanase + β-glucanase combination in pre-growout Nile tilapia (*Oreochromis niloticus*) [58]. Additionally, Wang et al. [62] reviewed the application of hemicellulases (xylanase and mannanase) in aquafeeds, focusing on their effects on nutrient digestion, immune response, and fish health. However, research specifically on NSP-degrading enzymes in FM-replaced aquafeeds remains extremely limited.

Current studies on single-enzyme supplementation in FM-replaced aquafeeds have focused on xylanase and cellulase, mostly in omnivorous fish. Supplementing 1,800–3,750 U/kg xylanase effectively mitigated the negative impacts of replacing 9%–30% FM with plant proteins (e.g., sunflower meal) in Nile tilapia (*Oreochromis niloticus*) [63] and mrigal (*Cirrhinus mrigala*) [64]. Notably, cellulase supplementation provided no additional growth benefits in crucian carp (*Carassius auratus*) fed diets replacing 53.2% FM with high-quality *Chlorella* meal (which already improved growth) [65]. In contrast, adding a mixed NSPase blend (Cellulase:Xylanase:Arabinoxylanase:Glucanase = 1:1:1:1) at 0.2% fully counteracted the negative effects of completely replacing 30% FM with rice distillers' dried grains soluble (DDGS) in climbing perch (*Anabas testudineus)* [66]. These findings underscore the urgent need for extensive research on single and composite NSPases in FM-replaced aquafeeds for a broader range of aquatic species, particularly carnivorous and herbivorous fish.

#### Phytase (PA)

Phytate (*myo*-inositol hexakisphosphate) is a ubiquitous ANF in plant protein ingredients. It reduces protein digestibility by forming indigestible protein–phytate complexes and directly inhibiting proteolytic enzymes (e.g., pepsin, trypsin) [67]. Dietary phytate also impairs intestinal structural and immune barriers, reduces antibacterial activity in the head kidney and spleen, and exacerbates inflammation, thereby suppressing growth in grass carp [68–70]. Phytases (*myo*-inositol-hexaphosphate phosphohydrolases) specifically hydrolyze phytate in plant-based aquafeeds. Monogastric animals lack endogenous phytase and cannot utilize phytate-bound nutrients. Research on phytase supplementation in FM-replaced aquafeeds is summarized in Table 3 and can be condensed into three trends: (1) Most studies focus on soybean protein sources (such as SBM and SPC); (2) Trials are limited mainly to juvenile carnivorous fish, with one study in shrimp; no data exist for species with other feeding habits; and (3) Supplementation of 1,000–2,000 FTU/kg phytase fully reverses growth depression caused by replacing 20%–25% FM with SBM/SPC in red sea bream and snakehead (*Channa striata*, *C. micropeltes*) [71–73]. However, phytase only partially alleviated growth inhibition at high FM replacement levels (53.5% by SPC) in sea bream (*Pagrus major*) [71] and only partially mitigated the negative effects of 10% FM replacement by SBM in Pacific white shrimp (*Penaeus vannamei*) at 2,152 FTU/kg [74]. Further research is warranted to evaluate phytase efficacy across diverse aquatic species and alternative plant protein sources.

**Table 3:** The application of exogenous enzymes in FM-replaced aquafeeds

Species	IBW, g	Period, d	**FM levels**		Alternative protein sources	NC vs. PC	**NC + Exogenous enzymes**				References
			PC	NC			Exogenous enzymes	Optimal levels	Compared with the NC group	Compared with the PC group	
NSPases											
Nile tilapia (*Oreochromis niloticus)*	1.31	84	18	9	Sunflower meal	FBW: ↓18%	Xylanase	3,750 U/kg	FBW: ↑16%	FBW: NS	[63]
Mori (*Cirrhinus mrigala*)	8.34	60	40	10	CSM 15%, Sunflower meal20%, CGM18.25%	FBW: ↓10%	Xylanase	1,800 U/kg	FBW: ↑16%	FBW: ↑4%	[64]
Crucian carp (*Carassius auratus)*	2.90	56	53.20	0	*Chlorella* meal	FBW: ↑74%	Cellulase	2,320 U/kg	FBW: NS	FBW: ↑75%	[65]
Climbing perch (*Anabas testudineus)*	5.0	60	30	0	Rice DDGS	PWG: ↓22%	NSPase (Cellulase:Xylanase: Arabinoxylanase:Glucanase = 1:1:1:1, w/w)	0.2%	PWG: ↑25%	PWG: NS	[66]
Phytase, FTU/kg diet											
Chinese sucker (*Myxocyrinus asiaticus*)	13	56	40	16	SBM	SGR: ↓13%	Phytase	1,500	SGR: ↑26%	SGR: NS	[42]
Sea bream (*Pagrus major*)	21	70	67	13.5	SPC	FBW: ↓25%	Phytase	2,000	FBW: ↑10%	FBW: ↓18%	[71]
Red seabream (*Pagrus major*)	24.0	42	65	40	SBM	FBW: ↓13%	Phytase	2,000	FBW: ↑15%	FBW: NS	[72]
Snakehead (*Channa striata* and *Channa micropeltes*)	4.74	56	59.67	35.8	SBM	FBW: ↓26%	Phytase	1,000	FBW: ↑23%	FBW: NS	[73]
Pacific white shrimp (*Penaeus vannamei*)	0.3	63	20	10	SBM	FBW: ↓15%	Phytase	2,152	FBW: ↑6%	FBW: ↓10% (Partial)	[74]
Protease, mg/kg diet											
Sea bass (*Dicentrarchus labrax)*	3.68	84	30	24	SBM	FBW: ↓9%	Papain (2,750 U/g)	500	FBW: ↑9%	FBW: NS	[75]
Gibel carp (*Carassius auratus gibelio)*	35.0	84	9	3	SBM	FBW: ↓17%	Protease (did not indicated enzyme activity)	150–175	FBW: ↑14%	FBW: NS	[76]
Pacific white shrimp (*Penaeus vannamei*)	0.3	56	20	10	SBM	FBW: ↓16%	Protease (81,800 U/g)	800	FBW: ↑11%	FBW: ↓7% (Partial)	[77]
Pacific white shrimp (*Penaeus vannamei*)	0.3	63	20	10	SBM	FBW: ↓15%	Protease (730,000 NFP/g)	50	FBW: ↑9%	FBW: ↓7% (Partial)	[74]
Complex enzymes, mg/kg diet											
Nile tilapia (*Oreochromis niloticus*)	6.30	90	11	6	CSJ	FBW: ↓9%	Protease, pepsin, trypsin, xylanase and amylase	500	FBW: ↑5%	FBW: NS	[78]

*PC* Positive control (FM control), *NC* Negative control (low-FM or FM-free), *IBW* Initial body weight, *FBW* Final body weight, *PWG* Percent weight gain, *SGR* Specific growth rate, *NS* No significant difference

#### Other exogenous enzymes and enzymes mixture

Other exogenous enzymes can also compensate for insufficient digestive capacity and improve non-FM protein utilization. Current research has focused on exogenous enzymes for soybean-dominated FM replaced aquafeeds. Supplementation with 500 mg/kg papain in European sea bass (*Dicentrarchus labrax*) [75] and 150–175 mg/kg protease to gibel carp (*Carassius auratus gibelio*) [76] mitigated the adverse effects of 6% FM replacement by SBM, restoring performance to FM control levels. However, in Pacific white shrimp, 800 mg/kg protease only partially alleviated growth suppression from 10% FM replacement by SBM [77]. Notably, a multi-enzyme blend (500 mg/kg, including protease, pepsin, trypsin, xylanase, and amylase) fully reversed the negative impacts of replacing 5% FM with a mixed plant protein blend (CSM, sunflower meal, jojoba meal) in Nile tilapia, improving growth, digestibility, physiology, and immunity [78]. However, other promising alternative proteins (insect meal, bacterial protein and algal protein) also contain indigestible components that limit their application. Thus, developing and validating targeted exogenous enzymes for these alternative protein sources is critically needed.

### Functional additives for health enhancement in FM-replaced aquafeeds

ANFs and indigestible components in alternative protein sources not only reduce nutrient digestibility but also damage intestinal and liver health, thereby impairing growth and metabolism. For example, SBM commonly induces intestinal inflammation and lipid metabolic disorders in fish [79]. Supplementing functional additives represents an effective strategy to alleviate these impairments and improve the utilization of alternative protein sources (Table 4).

**Table 4:** The application of functional additive in FM-replaced aquafeeds

Species	IBW, g	Period, d	**FM levels**		Alternative protein sources	NC vs. PC	**NC + Functional additives**				References
			PC	NC			Functional additive	Optimal levels	Compared with the NC group	Compared with the PC group	
Rainbow trout (*Oncorhynchus mykiss*)	13	70	48	0	SBM and CGM	FBW: ↓17%	Bile salts	1%	FBW: ↑15%	FBW: NS	[79]
Chinese soft‑shelled turtle	15	60	51	31	Poultry by‑product meal	SGR: ↓11%	Bile acid	0.1%	SGR:↑21%	SGR: NS	[80]
Pacific white shrimp (*Litopenaeus vannamei*)	0.25	50	25	10	SPC and dephenol cottonseed protein	FBW: ↓10%	Bile acid	900 mg g/kg	FBW: ↑7%	FBW: NS	[81]
Pacific white shrimp (*Litopenaeus vannamei*)	1.09	56	20	14	SBM	FBW: ↓10%	Bile acid	150 mg/kg	FBW: ↑11%	FBW: NS	[82]
*Penaeus monodon*	1.01	56	25	15	SPC	WG:↓	Chenodeoxycholic acid	0.1%	WG: ↑	WG: NS	[83]
Pacific white shrimp (*Litopenaeus vannamei*)	0.25	56	25	12.5	*Clostridium autoethanogenum* protein	FBW:↓7%	Chenodeoxycholic acid	0.06%	FBW: ↑4%	FBW: NS	[84]
Pacific white shrimp (*Litopenaeus vannamei*)	0.33	56	25	15	SPC	SGR: NS Serum GPT (liver injury): ↑44%	Chenodeoxycholic acid	0.08%	SGR: NS Serum GPT (liver injury): ↓29%	SGR: NS Serum GPT (liver injury): NS	[85]
Pacific white shrimp (*Litopenaeus vannamei*)	0.45	56	21	11	Tenebrio molitor meal	FBW: ↓20%	Ginseng saponins	60 mg/kg	FBW: ↑17%	FBW: ↓6% (Partial)	[86]
Japanese eel (*Anguilla japonica*)	9	56	65	52	FM analog^1^	SGR: ↓8%	Song-Gang stone	0.4%	SGR: ↑7%	SGR: NS	[87]
Japanese eel (*Anguilla japonica*)	9	56	65	52	FM analog^1^	SGR: ↓8%	Yucca meal	0.1%	SGR: ↑8%	SGR: NS	[87]
European sea bass (*Dicentrarchus labrax*)	37.3	60	22.5	10	Plant-based protein^2^	SGR: ↓17%	Commercial blend of anise, citrus, and oregano essential oils (Digestarom PEP M.G.E 150)	0.2 g/kg	SGR: ↑7%	SGR: ↓11%	[88]
Japanese seabass (*Lateolabrax japonicus*)	125.6	105	42	21	SBM	PWG: ↓	Mixture^3^	665 mg/kg	PWG: ↑	PWG: ↓(Partial relief)	[89]
Yellowtail kingfish (*Seriola lalandi*)	22	33	40	20	BSF meal^4^	SGR: NS	Garlic and tuna hydrolysate additives	4%	SGR: NS	SGR: NS	[90]
Pacific white shrimp (*Litopenaeus vannamei*)	3.03	63	24	2	PP	Survival: ↓50% FCR: ↑65%	Commercial phytogenic feed additive Digestarom PEP MGE	200	Survival: ↑65% FCR: ↓29%	Survival: NS FCR: NS	[91]

*PC* Positive control (FM control), *NC* Negative control (low-FM or FM-free), *IBW* Initial body weight, *FBW* Final body weight, *PWG* Percent weight gain, *SGR* Specific growth rate, *NS* No significant difference

^1^FM analog: 15% leather meal (The Feed Co., Seoul, Republic of Korea), 15% poultry by-product meal (Cherry Buro Co., Chungcheongbuk-do, Republic of Korea), 25% feather meal (Cherry Buro Co., Chungcheongbuk-do, Republic of Korea), 2% tuna by-product (Woonam Fish Co., Seoul, Republic of Korea), 24% blood meal (The Feed Co., Seoul, Republic of Korea), 1% squid liver powder (The Feed Co., Seoul, Republic of Korea), 15% soybean meal (The Feed Co., Seoul, Republic of Korea), 2% lysine (The Feed Co., Seoul, Republic of Korea), and 1% methionine (The Feed Co., Seoul, Republic of Korea)

^2^Plant-based protein: SPC, wheat gluten, CGM and SBM

^3^Mixture: ethoxyquin (0.02% Agrado^®^, Columbia), a 1:1 standardized combination of thymol and carvacrol (0.006% NE-150^®^) as well as chelated trace elements (0.0055% Mintrex^®^ Cu, 0.01% Mintrex^®^ Mn and 0.025% Mintrex^®^ Zn) (Novus International Inc., St. Charles, MO, USA)

^4^BSF meal: Partially defatted black soldier fly (*Hermetia illucens*) meal

#### Bile acids

Bile acids are among the most studied functional additives in FM-replaced aquafeeds, especially for crustaceans, which cannot synthesize bile acids endogenously. In Pacific white shrimp (*Litopenaeus vannamei*) and black tiger shrimp (*Penaeus monodon*), replacing 6%–15% FM with SBM, SPC, or *Clostridium autoethanogenum* protein reduced growth performance, but targeted supplementation of bile acids or chenodeoxycholic acid fully restored growth to the FM control level [81–85]. Similarly, bile acid supplementation reversed the negative effects of 20% FM replacement by poultry by-product meal in Chinese soft-shelled turtles [80]. In carnivorous fish, 1% bile salt supplementation completely alleviated growth inhibition caused by replacing 48% FM with SBM and CGM in rainbow trout [79]. These results demonstrate that bile acids are highly effective in both crustaceans and carnivorous fish. Further evaluation is needed across a wider range of aquatic species.

#### Phytochemicals and commercial functional additives

Several phytogenic and commercial additives have also been evaluated in FM-replaced aquafeeds. In Pacific white shrimp, the phytogenic feed additive *Digestarom* PEP MGE completely counteract the negative effects of replacing 22% FM with plant proteins [91], whereas ginseng saponins only partially mitigated growth depression caused by 10% FM replacement with *Tenebrio molitor* meal [86]. In carnivorous fish, dietary supplementation with song-gang stone (4 g/kg), yucca meal (1 g/kg), garlic, or tuna hydrolysate (4%) fully alleviated the adverse effects of 13%–20% FM replacement by plant or mixed proteins in Japanese eel (*Anguilla japonica*) and yellowtail kingfish (*Seriola lalandi*) [87, 90]. However, a commercial blend of anise, citrus, and oregano oils only partially alleviated growth inhibition in European seabass (*Dicentrarchus labrax*) and Japanese seabass (*Lateolabrax japonicus*) when 12.5%–21% FM was replaced by plant proteins or SBM [88, 89]. Together, current research remains concentrated on carnivorous fish and crustaceans. Further studies are required for omnivorous and herbivorous fish to support practical total FM replacement.

#### Prebiotics, plant extracts, short-chain fatty acids, and immune enhancers

Multiple other functional substances improve health and growth in low-FM or FM-free diets. Prebiotics (xylooligosaccharides and mannose) enhanced intestinal structure, immunity, feed intake, feed efficiency, and growth in grass carp [92–98]. Plant extracts such as cinnamaldehyde [99–101], fraxetin [102, 103] and tea polyphenols [104–107] produced similar beneficial effects. Additionally, curcumin [108, 109], 4-methylxanthone [110–112] and hydroxytyrosol [113, 114] reduced mycotoxicity, supporting safer use of mycotoxin‑prone plant proteins. Short-chain fatty acids (especially butyric acid) strongly improved intestinal health [115, 116]. Immune enhancers (nucleotides and antimicrobial peptides) regulated immune function and promoted growth in grass carp [117–121]. However, the specific efficacy of these additives in mitigating FM replacement stress remains to be systematically verified. Further research will provide critical support for reducing dietary FM levels in sustainable aquafeeds.

## Conclusion

**Fig. 1:**
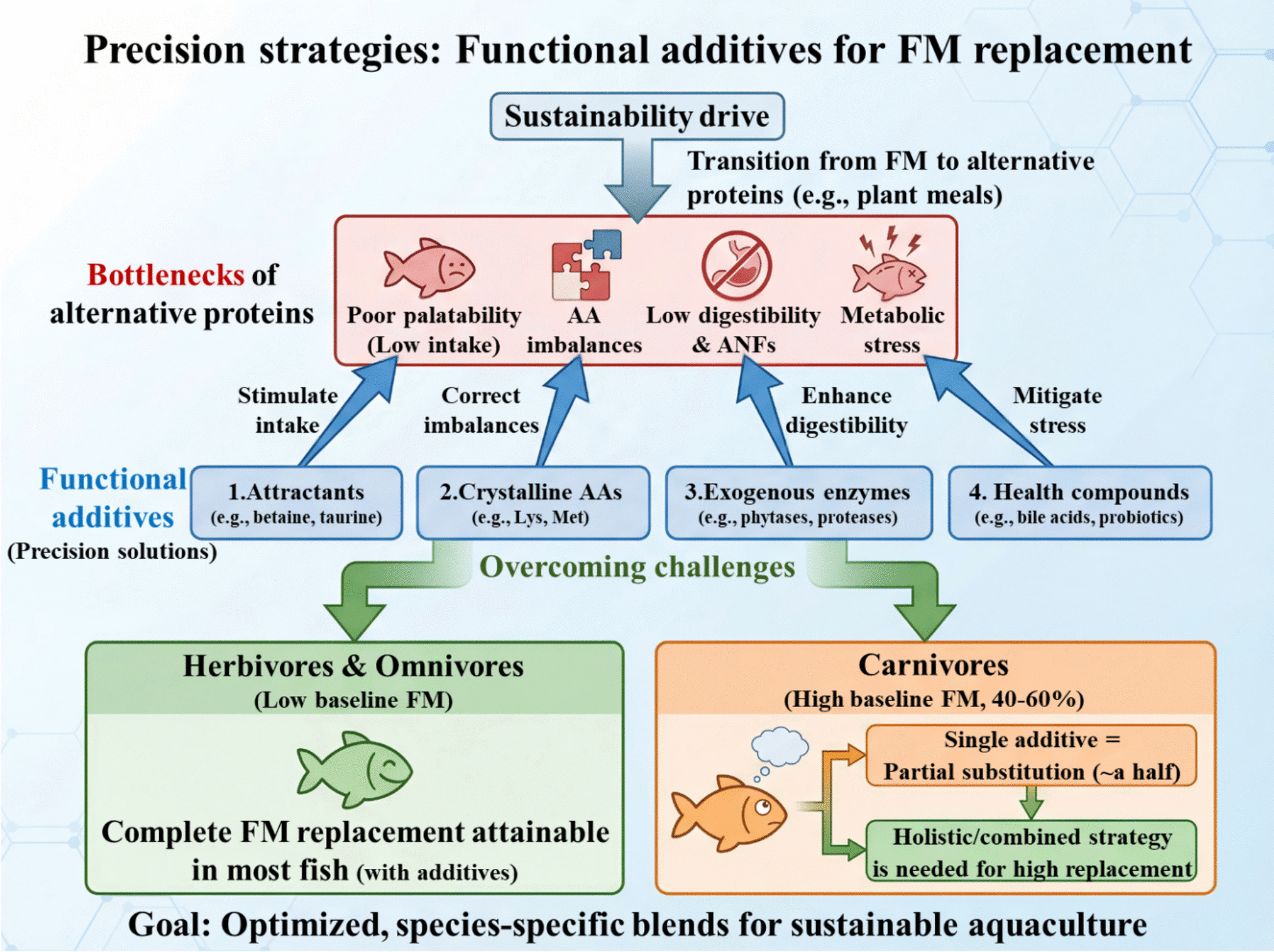
The application effect of functional additives in FM-replaced aquafeeds (This figure was drawn by the Nano Banana Pro-4 K software)

This review systematically evaluates the strategic use of functional additives to overcome challenges in FM-replaced aquafeeds (Fig. 1). While alternative proteins (e.g., plant meals) are crucial for sustainability, they often compromise fish growth due to poor palatability, AA imbalances, low digestibility, and ANFs. To mitigate these issues, functional additives are categorized into four key groups: (1) attractants (e.g., betaine, taurine, Br-DMPT) to stimulate feed intake; (2) crystalline AAs (mainly Lys and Met) to correct AA imbalances; (3) exogenous enzymes (e.g., phytases, proteases) to improve nutrient digestibility; and (4) health-promoting compounds (e.g., bile acids) to reduce metabolic stress. Their application in FM-replaced aquafeeds can be summarized as follows: (1) Herbivorous fish (primarily grass carp and blunt snout bream): Adding attractants such as taurine and Br-DMPT in grass carp or Lys in blunt snout bream fully alleviated the negative effects on FI and growth when SPC replaced 4%–5% of FM, enabling FM-free formulations. (2) Omnivorous fish (mainly Nile tilapia and carp species): For tilapia, single additives, including betaine, crystalline Lys, xylanase, or a composite enzyme mix, sufficed to offset growth impairments from partial or full FM replacement by plant proteins. In yellow river carp, an AA mixture or Br-DMPT only partially mitigated issues when 14% FM was replaced. For gibel carp, adding protease fully resolved the negative effects of replacing 6% FM with soybean meal. (3) Carnivorous fish (focus on rainbow trout, seabream, sea bass etc.): Given their high FM baselines (40%–60% of diet), single additives generally allow only partial substitution (≈50%). Higher replacement rates require combined strategies addressing palatability, AA balance, and digestive health simultaneously. (4) Crustaceans (focus on Pacific white shrimp): Functional additives appear to support only limited FM replacement (10%–15%) with alternative protein sources. Notably, when high-quality alternative protein sources were used without causing growth impairment, adding functional additives generally yielded no further improvement. In summary, developing cost-effective, species-specific additive blends is essential to advance sustainable aquaculture with reduced reliance on FM.

## Challenges and future perspectives

Increasing aquaculture production is essential to reach the Global Sustainable Development Goal #2 (Zero Hunger). The development of high quality aquafeeds with low or no FM is essential for further sustainable expansion of this sector. Fish and other aquatic animals, especially carnivorous, do not have an evolutionary adaptation to most of these non-FM feed ingredients. Therefore, a wide range of additives is needed to make the feeds palatable, balance nutrient requirements, reduce anti-nutritive substances and/or improve gut health. To advance sustainable aquaculture and reduce reliance on FM, future research should prioritize the following areas.

### Development of synergistic additive blends

Carnivorous species represent the primary demand for FM, with dietary inclusion levels approximately 2–3 times and 8–10 times greater than those for omnivorous and herbivorous species, respectively. Current evidence shows that while single additives can help alternative protein sources replace about half of the FM in carnivorous diets (where FM typically constitutes 40%–60% of the formulation), higher replacement rates require a combined additive strategy. Accordingly, future research should prioritize identifying optimal blends of attractants, crystalline amino acids, enzymes, and immunostimulants, tailored to the specific properties and limitations of alternative protein sources. The objective is to engineer synergistic effects that concurrently address multifaceted nutritional and physiological constraints.

### Species-specific precision formulations

Research must advance beyond generic additive approaches. There is a critical need to develop tailored formulations based on the specific metabolic and digestive physiology of each species. This is particularly important for carnivorous fish, for which a “one size fits all” solution is ineffective.

### Economic viability and cost-effectiveness

To facilitate industry adoption, future work should aim to optimize production methods for functional additives to lower costs. Furthermore, research must establish definitive economic thresholds where the expenditure on additives is counterbalanced by savings derived from reduced FM inclusion and augmented feed efficiency.

### Refinement of holistic nutritional strategies

For omnivorous and herbivorous species, where complete FM replacement is already feasible (at baseline inclusion levels of ~20% and ~5%, respectively), research should focus on sustaining long-term health and maintaining flesh quality under total FM exclusion. This ensures that advancements in sustainability do not compromise product quality.

## Data Availability

No datasets were generated or analysed during the current study.

## Abbreviations

AA: Amino acid
ANFs: Anti-nutritional factors
CGM: Corn gluten meal
CSJ: Cottonseed meal, sunflower meal, and jojoba meal
CSM: Cottonseed meal
DDGS: Distillers' dried grains soluble
EAA: Essential amino acids
FBW: Final body weight
FI: Feed intake
FM: Fishmeal
Lys: Lysine
Met: Methionine
MHA: Methionine hydroxy analogue
NSP: Non-starch polysaccharides
NSPase: Non-starch polysaccharide enzyme
PP: Plant protein
PUFA: Polyunsaturated fatty acids
PWG: Percent weight gain
RPC: Rice protein concentrate
RSM: Rapeseed meal
SBM: Soybean meal
SGR: Specific growth rate
SPC: Soy protein concentrate
Thr: Threonine
Trp: Tryptophan
WG: Weight gain
